# Role of vasoactive intestinal peptide in osteoarthritis

**DOI:** 10.1186/s12929-016-0280-1

**Published:** 2016-08-23

**Authors:** Wei Jiang, Hua Wang, Yu-sheng Li, Wei Luo

**Affiliations:** 1 Department of Bone and Joint, The Second Clinical Medical College (Shenzhen People’s Hospital), Jinan University, Shenzhen, Guangdong 518020 China; 2 Department of Orthopaedics, Xiang-ya Hospital, Central South University, Changsha, Hunan 410078 China

**Keywords:** Vasoactive intestinal peptide, Osteoarthritis, Cytokines, VIP receptors, Immuno-modulation

## Abstract

Vasoactive intestinal peptide (VIP) plays important roles in many biological functions, such as, stimulation of contractility in the heart, vasodilation, promoting neuroendocrine-immune communication, lowering arterial blood pressure, and anti-inflammatory and immune-modulatory activity. Osteoarthritis (OA) is a chronic and degenerative bone disease, which is one of the most common causes of disability and most common in both sexes as people become older. Interestingly VIP can prevent chronic cartilage damage and joint remodeling. This review article provides update information on the association of VIP and OA and its treatment. Evidences suggest that VIP is down-regulated in synovial fluid of OA, and VIP down-regulation leads to increase in the production of pro-inflammatory cytokines that might contribute to the pathogenesis of OA; however contradictory reports also exist suggesting that accumulation of VIP in joints can also contribute OA. A number of studies indicated that up-regulation of VIP can counteract the action of pro-inflammatory stimuli and alleviate the pain in OA. More clinical investigations are necessary to determine the biology of VIP and its therapeutic potential in OA that might represent the future standards of care for OA.

## Background

The vasoactive intestinal peptide (VIP) also known as the vasoactive intestinal polypeptide is a neuropeptide that belongs to a glucagon/secretin superfamily, the ligand of class II G protein-coupled receptors [1, 2]. It was initially reported to be expressed in lung and small intestine tissue; however, currently it is also described in neurons of central nervous system (CNS) [3, 4]. Beside the neuronal source, VIP is also expressed and released from endocrine organs (such as heart, thyroid, kidney and gastrointestinal tracts) and immune organs (such as spleen, thymus, bone marrow and lymph nodes) [5]. VIP plays essential roles in a broad spectrum of biological functions. It stimulates contractility in the heart, causes vasodilation, promotes neuroendocrine-immune communication, increases glycogenolysis and lowers arterial blood pressure [1, 3]. VIP displays potent anti-inflammatory and immune-modulatory activity and modulation of VIP level has considered being a potential candidate for treatment of inflammatory and autoimmune diseases including acute pancreatitis, septic shock, inflammatory bowel disease, lipopolysaccharide (LPS)-induced acute inflammation and arthritis [6, 7].

Osteoarthritis (OA) is a chronic and degenerative disease that results from the breakdown of articular cartilage components (particularly type II collagen and cartilage specific proteoglycans) and extensive remodeling of subchondral bone leading to synovitis as well as osteophyte formation [8, 9]. OA is the most common form of arthritis with disease of near the ends of the fingers, lower back, knees and hips which may affect weight bearing, mobility and normal daily activities [10]. It is one of the most common causes of disability and most common in both sexes as people become older. Among adults in the age of over 60 years, the prevalence of OA is approximately 10 % in men and 13 % in women [11]. The exact cause of OA is unclear. The etiology of OA is complex and multi-factorial including age, sex, and body weight, genetic, biological and biomechanical components. OA is believed to be caused by previous joint injury, abnormal limb development or mechanical stress on the joint [12, 13]. The most common clinical symptoms of OA are joint pain, swelling, decreased range of motion and stiffness. The joint pain is mainly due to the inflammation effects and the sensitization of neurobiological processes and further stimulation cascade of inflammation responses. During inflammation, mechanical stimuli sensitize joint nerves the actions of certain neuropeptides, eicosanoids, proteinase-activated receptors and ion channel ligands. Usually the inflammatory neuropeptides substance P, calcitonin gene-related peptide, as well as VIP, have all been reported to be immunolocalized in joint tissues and their levels are increased in arthritis [14].

Treatment of OA includes exercise, lifestyle modification and pain medications. The treatment of OA is still restricted to analgesics which have some undesirable and hazardous side effects. If the conventional management is ineffective and pain interferes with normal life, joint replacement surgery may be recommended. Evidence supports that joint replacement is both clinically effective and cost-effective; however, the functional and clinical outcomes can be poor [9, 12]. Therefore, alternative therapeutics is urgently needed that could improve the survival rate and quality of life of OA patients. A better understanding of the pathological mechanisms by which it promotes initiation and maintenance of joint pain in OA may help to identify the more effective targets to counteract the debilitating symptoms of OA [15, 16].

Evidence suggests that VIP is shown to prevent chronic cartilage damage and joint remodeling [7]. It shows the therapeutic function in rheumatoid arthritis (RA); however, the protective function of VIP in the progression of OA is not fully explained [2, 17]. In this review, we have focused on the association of VIP in the development and progression of OA and the potential effects of VIP as a therapeutic agent for the treatment of OA.

## Review

### Basic structure and functions of VIP

VIP is a widely distributed neuropeptide that functions as a neurotransmitter or neuromodulator in many organs and tissues. It contains 28 amino acid residues and in humans, it is encoded by the *VIP* gene [1, 18]. The gene is approximately 9 kb long and contains 7 exons, each encoding a distinct functional domain. Exon 1 consists of the 5’-untranslated region of the mRNA, exon 2 encodes a putative signal peptide, exon 3 encodes an N-terminal peptide, exon 4 encodes PHM (peptide with N-terminal histidine and C-terminal methionine amide), exon 5 encodes VIP, exon 6 encodes the C-terminal peptide and exon 7 consists of the 3’-untranslated region of the mRNA [4, 19].

VIP consists of 3 receptors: VIP receptor type 1 (VPAC1), VIP receptor type 2 (VPAC2) and pituitary adenylate cyclase-activating polypeptide (PACAP) type 1 (PAC1) receptor [20, 21]. These receptors belong to family 2 of the G protein–coupled receptors and elicit remarkable anti-inflammatory and immune-modulatory properties by up-regulating cyclic adenosine 5’-phosphate (cAMP), adenylate cyclase and phospholipase C (PLC) [2, 9]. VPAC receptors are expressed in macrophages as well as in lymphocytes among which VPAC1 is thought to be the major receptor type present during early culture period while VPAC2 is expressed relatively late during macrophage/monocyte activation as an inducible receptor after T cell receptor triggering or LPS stimulation. VPAC receptors are expressed in macrophages as well as in lymphocytes among which VPAC1 is thought to be the major receptor type present during early culture period while VPAC2 is expressed relatively late during macrophage/monocyte activation as an inducible receptor after T cell receptor triggering or LPS stimulation. PAC1 was found to be expressed in cells of the macrophage/monocyte lineage. The differential expression of VIP receptors (VPAC1 and VPAC2) in arthritis is also reported. The expression of VPAC1 mRNA is significantly decreased while the expression of VPAC2 mRNA is increased in arthritic fibroblast-like synoviocytes (FLS) [5, 17].

VIP is synthesized from a precursor molecule (prepro-VIP) which is metabolized by a signal peptidase in the endoplasmic reticulum to yield pro-VIP. The pro-VIP is cleaved by prohormone convertases to form VIP-GKR (prepro-VIP125–155) which is then cleaved by carboxypeptidase-B-like enzymes to VIP-G (glycine-extended VIP). The VIP-G can then be metabolized by PAM enzymes to VIP [4].

VIP has pleiotropic effects as a neurotransmitter, immune regulator, vasodilator and secretagogue. In CNS, VIP has been shown to have neurotrophic effects that affect learning and behavior capacity [5]. VIP regulates bone metabolism, circadian rhythms and embryonic development, and plays an important role as immune modulator [5]. In focus on controlling joint inflammation, VIP plays dual role, as it functions as a macrophage deactivating factor which inhibits the production of the pro-inflammatory cytokines (IL-1, IL-6, IL-12) and tumor necrosis factor-α (TNF-α), while stimulates the production of anti-inflammatory cytokines such as IL-4, IL-10, IL-13 and insulin-like growth factor 1 (IGF-1) [4, 22]. It shifts the Th1 (pro-inflammatory)/Th2 (anti-inflammatory) balance by promoting TH2 differentiation and inhibiting TH1 responses and shows a protective effect on joint swelling, and destruction of cartilage and bone [7, 23]. VIP also modulates IL-22R1 expression and prevents the contribution of FLS in RA to counterbalance IL-22 effects on FLS behavior [24]. VIP also has significant effects on the cardiovascular system, where it contributes to the regulation of normal coronary vasomotor tone, increases the epicardial coronary artery cross-sectional area, decreases coronary vascular resistance and significantly increases coronary artery blood flow and vasodilation [18]. As a secretagogue, VIP induces release of various types of hormones including prolactin, luteinizing hormone and growth hormone from the pituitary, and regulates the release of insulin and glucagon in the pancreas [5].

### Association of VIP with OA

The association of VIP is more clearly detected in RA but the exact role of VIP in the pathogenesis of OA remains largely unknown [8]. In most of the studies, VIP functions in joint protection were performed in RA model; however, the protective function in the progression of OA is uncertain. Jiang et al. [2] suggested in their study that VIP may play a protective role in progression of OA just like in RA, as the expression of VIP in synovial fluid and articular cartilage from patients with OA is negatively associated with progressive joint damage and disease sevrity.

**Fig. 1:**
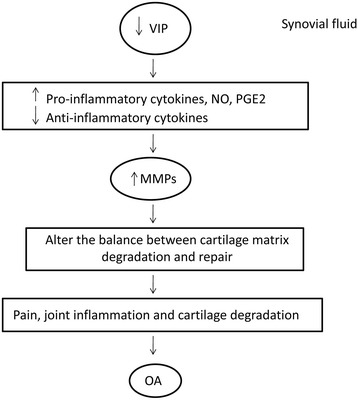
Negative role of VIP in osteoarthritis pathogenesis. The expression of VIP is down-regulated in synovial fluid of OA and down-regulation of VIP stimulates the production of pro-inflammatory cytokines that might contribute to the pathogenesis of OA by developing pain, joint inflammation and cartilage degradation [16, 25, 33]

VIP is reported to be localized in postganglionic sympathetic and sensory nerve fibres that are in close proximity to the synovial fluid and serum of arthritis patients. FLS are abundant synovial cell populations that play regulatory roles in inflammation and joint destruction. Synovial thickening is frequently present in OA and uniquely associated with the severity of knee pain [25]. VIP expression is down-regulated in synovial fluid and articular cartilage of OA patients, and down-regulation of this endogenous VIP might contribute to the pathogenesis of OA by providing contribution significantly to the development of pain, joint inflammation and cartilage degradation (Fig. 1) [2, 17]. In OA FLS, endogenous VIP is found at a much lower level. Lower level of endogenous VIP stimulates the formation of various catabolic mediators including pro-inflammatory cytokines, nitric oxide and prostaglandin E2, and contributes to the pathogenesis of OA by altering the balance of cartilage matrix degradation and repair [26]. VIP also stimulates nuclear translocation of cytokine transcription factors (NFkB and c-JUN) and subsequent TNF-α production by human monocytes through the reduction of LPS, and up-regulation of the TLR-2 and TLR-4 Toll-like receptors [27–29]. VIP suppresses the phosphorylation of IKKβ, prevents degradation of the IkBα and inhibits NFkB binding activity, thereby, modulates cell function [30]. VIP impairs the development of self reactive Th1 and Th17 cells, activates macrophages and neutrophils as well as increases the production of pro-inflammatory and joint destruction mediators such as CCL2 (MCP-1, monocyte chemotactic protein 1), CXCL8 [interleukin (IL)-8], IL-6, interferon-β (IFN-β) and IFN regulatory factor 3 (IRF-3) in OA FLS [31–33]. The expression of the urokinase plasminogen activator (uPA) system components is increased in OA. In the presence of lower level of VIP, IL-1β as inflammatory cytokine, and Fn fragments derived from cartilage destruction stimulated uPA system and promotes the perpetuation of the destructive cascade in joint through the production of matrix metalloproteinases-9 (MMP-9) and metalloproteinases-13 (MMP-13) in OA-FLS [34–36].

**Fig. 2:**
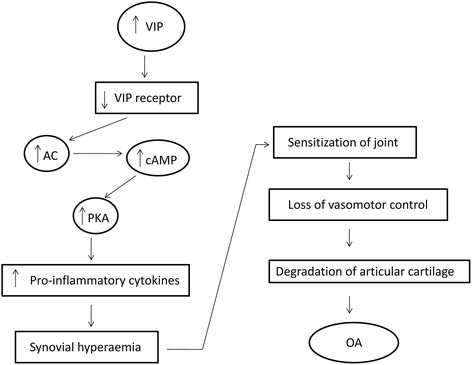
Positive role of VIP in osteoarthritis pathogenesis. VIP is released into OA knee joints and subsequent accumulation of VIP in the joint down-regulates the VPAC receptor. Up-regulation of VIP increases the production of pro-inflammatory cytokines which in turn sensitizes afferent nerve fibres leading to the vascular dysfunction and degenerative changes in OA [37, 38, 40]

One contradictory study has suggested that up-regulation of VIP in arthritic knees induces synovial hyperaemia via VPAC receptors located within the knee joint and sensitization of joint afferents leading to nociception. The mechanism by which VIP exerts its pro-nociceptive effects includes the enhanced production of cAMP and enhanced protein kinase A activity through the stimulation of adenylyl cyclase [37]. VIP is released into OA knee joints and sensitizes afferent nerve fibres, enhanced firing rate during joint movement and reduced mechanical threshold after induction of OA [38]. It is reported that stimulation of synovial VPAC receptors by VIP in OA knees increases the production of IL-1β, IL-6 and TNF-α which in turn sensitize joint afferent nerve terminals leading to induce mechanosensitivity and pain [9]. Subsequent accumulation of VIP in the joint down-regulates the VPAC receptor and the vasodilatatory effect of VIP is lost during acute knee joint inflammation. VIP-containing nerves have the potential to involve in articular vasoregulation. VIP-induced vasomotor control is found to be altered in arthritis [39, 40]. Mast cells are involved in modulating tissue inflammation and activation of these immune cells results in cellular degranulation. The inflammatory mediators are released into the extracellular environment contributing vascular dysfunction and alter the homeostatic mechanisms that regulate synovial blood flow in OA. As synovial fluid provides nutrition to articular cartilage, loss of vasomotor control of the synovial microcirculation could contribute to the degenerative changes in OA (Fig. 2) [41, 42].

VIP level is actually down-regulated in both synovial fluid and areticular cartilage, and the level is negatively correlated with disease severity [2]. However, studies found that extremely higher level of VIP accumulation in joint is responsible for receptor desensitization, and in a different mechanism, VIP contributes the production of pro-inflammatory cytokines. Thus, down-regulated VIP in synovial fluid and up-regulated VIP in joint are responsible for OA pathogenesis.

### Can VIP be used as target in OA?

OA is the most common form of arthritis and has become a big threat for normal healthy life. The conventional therapy of OA is still unsatisfactory as these are highly expensive and cannot cure totally. Effective prevention and therapeutic treatment of OA will require the identification of appropriate mediators of joint pain and articular cartilage degradation [43]. VIP, a natural protein with well-known physiological effects is being considered as a potential therapeutic agent and a promising candidate for the treatment of OA [8, 32]. It is reported that pro-inflammatory cytokines stimulate the production of matrix metalloproteinase and administration of VIP is reported to counteract the action of these pro-inflammatory stimuli and reduces the constitutive expression of MMP-9 and MMP-13, thereby, alleviating pain in OA [8, 34]. Pérez-García et al. [44] analyzed the effect of VIP on the corticotropin-releasing factor (CRF) system and urocortins (UCN1, UCN2, UCN3) in human OA synovial fibroblasts. VIP treatment in OA FLS causes a significant increase of the potential anti-inflammatory mediators (UCN2 and UCN3) through the modulation of the expression of CRF system that indicates the anti-inflammatory function and therapeutic potential of VIP in OA. In one study, Delgado et al. [7] reported that administration of VIP directly into the joints shown completely ameliorate collagen destruction and joint remodeling in a murine model of collagen-induced arthritis.

However, some contradictory studies reported that VIP is released into OA knee joints and potentially contributing to joint pain [9, 38, 45], and VIP inhibition may be a useful means of controlling joint pain. Treatment of OA knees with a VPAC receptor antagonist VIP6-28 significantly inhibits the sensitizing effect of VIP, decreases afferent firing rate and reduces mechanonociception in OA joints [9]. There is a lack of clinical evidence which may support that targeting VIP is successful in OA treatment. More investigations including in vivo studies or VIP knockout animal model are necessary to define the signaling events induced by VIP and its therapeutic potential in OA.

## Conclusion

OA is a chronic and debilitating disease among the elderly people. It is a complex and painful disease whose progression is associated with a chronic synovitis, degradation of articular cartilage, remodeling of subchondral bone and osteophyte formation. Currently, there are no satisfying drugs and agents for treating OA. Only management is possible to alleviate its symptoms. Despite the development of modern medical science, very little is known about the factors responsible for the pathogenesis of OA. Proper investigation of the modulating factors that might alter the incident of pain may improve the potential therapy for OA. VIP is reported as an anti-inflammatory and immune-modulatory peptide that has considered being a potential candidate for treatment of inflammatory and autoimmune diseases through down-regulation of inflammatory cytokines and mediators. However, only a few experiments have been conducted to demonstrate the potential value of VIP in the treatment of OA. More investigations are necessary to determine the biology of VIP and its therapeutic potential in OA that might represent the future standards of care for OA.

## Abbreviations

cAMP: Cyclic adenosine 5’-phosphate
CNS: Central nervous system
FLS: Fibroblast-like synoviocytes
LPS: Lipopolysaccharide
MMP: Matrix metalloproteinase
OA: Osteoarthritis
PAC1: Pituitary adenylate cyclase-activating polypeptide type 1 receptor
PACAP: Pituitary adenylate cyclase-activating polypeptide
PHM: peptide with N-terminal histidine and C-terminal methionine amide
RA: Rheumatoid arthritis
TLR: Toll-like receptor
TNF-α: Tumor necrosis factor-α
VIP: Vasoactive intestinal peptide
VPAC1: VIP receptor type 1
VPAC2: VIP receptor type 2
VPAC3: VIP receptor type 3

## References

[1] Umetsu Y, Tenno T, Goda N, Shirakawa M, Ikegami T, Hiroaki H (1814). Structural difference of vasoactive intestinal peptide in two distinct membrane mimicking environments. Biochim Biophys Acta.

[2] Jiang W, Gao SG, Chen XG, Xu XC, Xu M, Luo W (2012). Expression of synovial fluid and articular cartilage VIP in human osteoarthritic knee: a new indicator of disease severity?. Clin Biochem.

[3] Buljevic S, Detel D, Pucar LB, Mihelic R, Madarevic T, Sestan B (2013). Levels of dipeptidyl peptidase IV/CD26 substrates neuropeptide Y and vasoactive intestinal peptide in rheumatoid arthritis patients. Rheumatol Int.

[4] Delgado M, Pozo D, Ganea D (2004). The significance of vasoactive intestinal peptide in immunomodulation. Pharmacol Rev.

[5] Delgado M, Ganea D (2013). Vasoactive intestinal peptide: a neuropeptide with pleiotropic immune functions. Amino Acids.

[6] Gutierrez-Cañas I, Juarranz MG, Santiago B, Arranz A, Martinez C, Galindo M (2006). VIP down-regulates TLR4 expression and TLR4-mediated chemokine production in human rheumatoid synovial fibroblasts. Rheumatology.

[7] Delgado M, Abad C, Martinez C, Leceta J, Gomariz RP (2001). Vasoactive intestinal peptide prevents experimental arthritis by down-regulating both autoimmune and inflammatory components of the disease. Nat Med.

[8] Sutton S, Clutterbuck A, Harris P, Gent T, Freeman S, Foster N (2009). The contribution of the synovium, synovial derived inflammatory cytokines and neuropeptides to the pathogenesis of osteoarthritis. Vet J.

[9] Schuelert N, McDougall JJ (2006). Electrophysiological evidence that the vasoactive intestinal peptide receptor antagonist VIP6-28 reduces nociception in an animal model of osteoarthritis. Osteoarthritis cartilage.

[10] Berenbaum F (2013). Osteoarthritis as an inflammatory disease (osteoarthritis is not osteoarthrosis!). Osteoarthritis Cartilage.

[11] Zhang Y, Jordan JM (2010). Epidemiology of osteoarthritis. Clin Geriatr Med.

[12] Glyn-Jones S, Palmer AJ, Agricola R, Price AJ, Vincent TL, Weinans H (2015). Osteoarthritis. Lancet.

[13] Brandt KD, Dieppe P, Radin E (2009). Etiopathogenesis of osteoarthritis. Med Clin North Am.

[14] McDougall JJ (2006). Arthritis and Pain. Neurogenic origin of joint pain. Arthritis Res Ther.

[15] McDougall JJ (2006). Pain and OA. J Musculoskelet Neuronal Interact.

[16] Niissalo S, Hukkanen M, Imai S, Törnwall J, Konttinen YT (2002). Neuropeptides in experimental and degenerative arthritis. Ann N Y Acad Sci.

[17] Juarranz Y, Gutierrez-Canas I, Santiago B, Carrion M, Pablos JL, Gomariz RP (2008). Differential expression of vasoactive intestinal peptide and its functional receptors in human osteoarthritic and rheumatoid synovial fibroblasts. Arthritis Rheum.

[18] Henning RJ, Sawmiller DR (2001). Vasoactive intestinal peptide: cardiovascular effects. Cardiovasc Res.

[19] Tsukada T, Horovitch SJ, Montminy MR, Mandel G, Goodman RH (1985). Structure of the human vasoactive intestinal polypeptide gene. DNA.

[20] Gomariz RP, Juarranz Y, Abad C, Arranz A, Leceta J, Martinez C (2006). VIP-PACAP system in immunity: new insights for multitarget therapy. Ann N Y Acad Sci.

[21] Abad C, Martinez C, Leceta J, Gomariz RP, Delgado M (2001). Pituitary adenylate cyclase-activating polypeptide inhibits collagen-induced arthritis: an experimental immunomodulatory therapy. J Immunol.

[22] Chapman CR, Tuckett RP, Song CW (2008). Pain and stress in a systems perspective: reciprocal neural, endocrine, and immune interactions. J Pain.

[23] Juarranz Y, Abad C, Martinez C, Arranz A, Gutierrez-Cañas I, Rosignoli F (2005). Protective effect of vasoactive intestinal peptide on bone destruction in the collagen-induced arthritis model of rheumatoid arthritis. Arthritis Res Ther.

[24] Carrión M, Juarranz Y, Seoane IV, Martínez C, González-Álvaro I, Pablos JL (2014). VIP modulates IL-22R1 expression and prevents the contribution of rheumatoid synovial fibroblasts to IL-22-mediated joint destruction. J Mol Neurosci.

[25] Hill CL, Gale DG, Chaisson CE, Skinner K, Kazis L, Gale ME (2001). Knee effusions, popliteal cysts, and synovial thickening: association with knee pain in osteoarthritis. J Rheumatol.

[26] von Rechenberg B, McIlwraith CW, Akens MK, Frisbie DD, Leutenegger C, Auer JA (2000). Spontaneous production of nitric oxide (NO), prostaglandin (PGE2) and neutral metalloproteinases (NMPs) in media of explant cultures of equine synovial membrane and articular cartilage from normal and osteoarthritic joints. Equine Vet J.

[27] Arranz A, Gutiérrez-Cañas I, Carrión M, Juarranz Y, Pablos JL, Martínez C (2008). VIP reverses the expression profiling of TLR4-stimulated signaling pathway in rheumatoid arthritis synovial fibroblasts. Mol Immunol.

[28] Foster N, Cheetham J, Taylor JJ, Preshaw PM (2005). VIP Inhibits Porphyromonas gingivalis LPS-induced immune responses in human monocytes. J Dent Res.

[29] Juarranz Y, Gutiérrez-Cañas I, Arranz A, Martínez C, Abad C, Leceta J (2006). VIP decreases TLR4 expression induced by LPS and TNF-alpha treatment in human synovial fibroblasts. Ann N Y Acad Sci.

[30] Ding W, Wagner JA, Granstein RD (2007). CGRP, PACAP, and VIP modulate Langerhans cell function by inhibiting NF-kappaB activation. J Invest Dermatol.

[31] Carrión M, Juarranz Y, Pérez-García S, Jimeno R, Pablos JL, Gomariz RP (2011). RNA sensors in human osteoarthritis and rheumatoid arthritis synovial fibroblasts: immune regulation by vasoactive intestinal peptide. Arthritis Rheum.

[32] Juarranz MG, Santiago B, Torroba M, Gutierrez-Cañas I, Palao G, Galindo M (2004). Vasoactive intestinal peptide modulates proinflammatory mediator synthesis in osteoarthritic and rheumatoid synovial cells. Rheumatology (Oxford).

[33] Carrión M, Pérez-García S, Jimeno R, Juarranz Y, González-Álvaro I, Pablos JL (2013). Inflammatory mediators alter interleukin-17 receptor, interleukin-12 and −23 expression in human osteoarthritic and rheumatoid arthritis synovial fibroblasts: immunomodulation by vasoactive intestinal Peptide. Neuroimmunomodulation.

[34] Perez Garcia S, Carrión M, Jimeno R, Ortiz AM, González-Álvaro I, Fernández J (2014). Urokinase plasminogen activator system in synovial fibroblasts from osteoarthritis patients: modulation by inflammatory mediators and neuropeptides. J Mol Neurosci.

[35] Long DL, Willey JS, Loeser RF (2013). Rac1 is required for matrix metalloproteinase 13 production by chondrocytes in response to fibronectin fragments. Arthritis Rheum.

[36] Little CB, Barai A, Burkhardt D, Smith SM, Fosang AJ, Werb Z (2009). Matrix metalloproteinase 13-deficient mice are resistant to osteoarthritic cartilage erosion but not chondrocyte hypertrophy or osteophyte development. Arthritis Rheum.

[37] Hernanz A, Medina S, de Miguel E, Martín-Mola E (2003). Effect of calcitonin gene-related peptide, neuropeptide Y, substance P, and vasoactive intestinal peptide on interleukin-1beta, interleukin-6 and tumor necrosis factor-alpha production by peripheral whole blood cells from rheumatoid arthritis and osteoarthritis patients. Regul Pept.

[38] Hunter DJ, McDougall JJ, Keefe FJ (2008). The symptoms of osteoarthritis and the genesis of pain. Rheum Dis Clin North Am.

[39] McDougall JJ, Barin AK, McDougall CM (2004). Loss of vasomotor responsiveness to the mu-opioid receptor ligand endomorphin-1 in adjuvant monoarthritic rat knee joints. Am J Physiol Regul Integr Comp Physiol.

[40] McDougall JJ, Karimian SM, Ferrell WR (1995). Prolonged alteration of vasoconstrictor and vasodilator responses in rat knee joints by adjuvant monoarthritis. Exp Physiol.

[41] McDougall JJ, Barin AK (2005). The role of joint nerves and mast cells in the alteration of vasoactive intestinal peptide (VIP) sensitivity during inflammation progression in rats. Br J Pharmacol.

[42] Groneberg DA, Welker P, Fischer TC, Dinh QT, Grützkau A, Peiser C (2003). Down-regulation of vasoactive intestinal polypeptide receptor expression in atopic dermatitis. J Allergy Clin Immunol.

[43] Cheng C, Gao S, Lei G (2014). Association of osteopontin with osteoarthritis. Rheumatol Int.

[44] Pérez-García S, Juarranz Y, Carrión M, Gutiérrez-Cañas I, Margioris A, Pablos JL (2011). Mapping the CRF-urocortins system in human osteoarthritic and rheumatoid synovial fibroblasts: effect of vasoactive intestinal peptide. J Cell Physiol.

[45] McDougall JJ, Watkins L, Li Z (2006). Vasoactive intestinal peptide (VIP) is a modulator of joint pain in a rat model of osteoarthritis. Pain.

